# “Seeking a Bridge Over Troubled Waters” Older Men’s Experiences of the Transition from Spouse to Long-Term Caregiver for a Female Partner Living with Dementia—A Meta-Synthesis

**DOI:** 10.1177/23333936251379273

**Published:** 2025-10-02

**Authors:** Tina Sjøvoll, Anne Clancy, Gunn-Mari Holdø, Gabriele Kitzmüller

**Affiliations:** 1 Narvik Municipality, Norway; 2 The Arctic University of Norway, Tromsø, Norway

**Keywords:** dementia care, male caregivers, older adults, gender roles, mental health, qualitative research, meta-synthesis, demensomsorg, mannlige omsorgsgivere, eldre voksne, kjønnsroller, psykisk helse, kvalitativ forskning, metasyntese

## Abstract

There is a growing body of qualitative research on older men caring for a wife or female partner with dementia at home. However, little is known about their experience transitioning from husband to caregiver. This study aimed to summarize and interpret qualitative research to illuminate this transition. Using Sandelowski and Barroso’s meta-synthesis approach, we systematically searched five databases (2000–2024) and included 18 studies from nine countries. Findings were expressed through the overarching metaphor: “*Seeking a bridge over troubled waters*,” encompassing three main categories: (1) from partner to caregiver: adjusting to a new normal; (2) from connection to solitude: navigating the loss of companionship and social participation, and (3) the journey toward embracing a caregiver identity. Although committed to care the men struggled with unfamiliar roles, loneliness, and conflicting emotions. Despite longing for support, many showed resilience in taking on their new role. For some, caregiving led to personal growth, deeper self-understanding, and a renewed sense of meaning. Transitional experiences were consistent across cultures. Nurses can play a vital role in identifying barriers and resources, offering support, and helping older caregivers develop coping strategies. Access to respite and outreach services may help overcome older male caregivers’ reluctance to seek help.

## Sammendrag

Det finnes en økende mengde kvalitativ forskning om eldre menn som tar seg av en kone eller kvinnelig partner med demens i hjemmet. Likevel vet vi lite om deres erfaringer med overgangen fra ektefelle til omsorgsgiver. Denne studien hadde som mål å oppsummere og tolke kvalitativ forskning for å belyse denne overgangen. Ved å bruke Sandelowski og Barrosos metasyntese tilnærming gjennomførte vi et systematisk søk i fem databaser (2000–2024) og inkluderte 18 studier fra ni land. Funnene ble uttrykt gjennom den overordnede metaforen: «Å søke en bro over urolige farvann», som omfattet tre hovedkategorier: (1) Fra partner til omsorgsgiver: å tilpasse seg en ny hverdag; (2) Fra tilknytning til ensomhet: å navigere tapet av fellesskap og sosial deltakelse; og (3) Å omfavne en ny identitet som omsorgsgiver. Selv om mennene var dedikert til å gi omsorg, slet de med ukjente roller, ensomhet og motstridende følelser. Til tross for et sterkt behov for støtte, viste mange motstandskraft i møte med sin nye rolle. For noen førte omsorgsrollen til personlig vekst, dypere selvforståelse og en fornyet følelse av mening. Overgangserfaringene var konsistente på tvers av kulturer. Sykepleiere kan spille en viktig rolle i å identifisere barrierer og ressurser, tilby støtte og hjelpe eldre omsorgsgivere med å utvikle mestringsstrategier. Tilgang til avlastning og oppsøkende tjenester kan bidra til å overvinne eldre mannlige omsorgsgiveres motvilje mot å søke hjelp.

## Introduction

This study explores older male caregivers’ transitions when caring for a female partner living with dementia. Worldwide about 55 million people have dementia, with approximately ten million new cases annually, and this number is likely to reach 75 million by 2030, and 132 million by 2050 (Prince et al., 2015). Traditionally, women have been the main informal care providers for older people (Baker & Robertson, 2008). An aging population, women’s higher risk of dementia and men’s increased life expectancy suggest that the number of husbands caring for their wives with dementia at home will increase (Robinson et al., 2014). In the UK, the profile of carers is changing with age, as the number of male caregivers aged over 85 already exceeds that of female carers (Greenwood & Smith, 2015).

Overall, caregiving for patients with dementia is associated with significantly increased risk of depressive symptoms, major depressive disorder, anxiety, insomnia, hypertension, pain, and diabetes compared to non-caregivers (Laks et al., 2016). Sallim et al. (2015) found the odds of having depression being 2.51 times higher for spousal dementia caregivers. Nevertheless, recent studies highlight that positive aspects of dementia caregiving are evident and deserve greater attention, as they can support caregivers in adapting to their roles and enhancing their overall well-being (Cheng et al., 2016; Ponsoda & Díaz, 2024; Wang et al., 2022; Yu et al., 2018). Dementia caregiving can offer positive outcomes, including a sense of achievement, mutual connection, stronger family bonds, and personal growth (Yu et al., 2018). These benefits are supported by recognition of the caregiving role, adaptive thinking, and environments that foster meaning-making (Yu et al., 2018).

Gender differences in dementia caregivers’ experiences are evident, with men experiencing higher degrees of benefits related to the caregiver role (Ponsoda et al., 2023) and less strain than women (Connors et al., 2020; Robinson et al., 2014). Male caregivers show higher scores in terms of resilience (Ponsoda et al., 2023) and report higher levels of sense of coherence (Childers, 2019) protecting them against psychological distress and caregiver strain (del-Pino-Casado et al., 2019; Ponsoda et al., 2023). Male dementia caregivers show lower scores on overall depression levels than females (Pillemer et al., 2018). Men may experience different depressive symptoms than those included in the current diagnostic criteria and their expressions of sadness and emotional vulnerability have been perceived as less acceptable due to social norms (Martin et al., 2013). Sleep disturbances, back pain, irritation, and helplessness are frequently reported in male dementia caregivers (Martínez-Santos et al., 2021). For male caregivers there is a negative correlation between caregiver strain and the perceived quality of life of the care recipient (Conde-Sala et al., 2010).

Loneliness and isolation are common features of older male dementia caregivers’ experiences (Fee et al., 2023; Hammar et al., 2021). This may be due to the loss of spousal companionship, reluctance to leave the spouse alone, attend social gatherings, or accept professional help (Fee et al., 2023). Loneliness may be worse for older men as their limited social networks seem to diminish further (Robinson et al., 2014). For older male dementia caregivers, it is important to maintain couple hood as long as possible (Brown et al., 2007), and they seem more concerned about being separated from their spouse than female caregivers (Perren et al., 2007). The decline of sexual and emotional intimacy impacts male caregivers’ well-being negatively, leaving them sad and frustrated (Fee et al., 2021).

Men are influenced by traditional masculine roles and are more task-oriented than emotionally oriented in their caregiver role (Hong & Coogle, 2016; Kenny et al., 2020; Robinson et al., 2014). While male caregivers generally seek support sooner and more often than female caregivers (del Río-Lozano et al., 2013), male dementia caregivers seem reluctant to seek help (Greenwood & Smith, 2015; LaManna et al., 2024; Robinson et al., 2014) and struggle unsupported (Finn & Boland, 2021). Additionally, they hesitate to plan for respite care for the ill spouse (Poisson et al., 2023). Inadequate support services are often reported in studies exploring male dementia caregivers’ experiences (LaManna et al., 2024; Robinson et al., 2014).

Older men’s roles within the family have been shaped by longstanding gender norms, where caregiving for children, aging parents, and household responsibilities has traditionally been viewed as women’s domain. Therefore, the transition into the caregiver role poses challenges to men (Boyle, 2014; Bueno, 2025). Robinson et al. (2014) found that men rearticulated traditional masculine ideals to sustain their gender identity and legitimize participation in feminine roles.

Entering the role of a family caregiver has been described as a challenging transition (Adams, 2006). Although several reviews on older male caregivers in dementia exist (Fee et al., 2020; Greenwood & Smith, 2015; Nel & Board, 2019), the transitional experiences of this group remain unexplored. Research concerning older male dementia caregivers’ experiences has been described as sparse (Greenwood & Smith, 2015; Milligan & Morbey, 2016), though a growing body of qualitative studies is emerging. To our knowledge, no recent meta-synthesis has focused specifically on older male caregivers’ transitions. Therefore, the aim of this study was to summarize, synthesize, and interpret qualitative research on older men’s experiences of transitions as caregivers for a wife or female partner living with dementia at home.

## Method

According to Sandelowski and Barroso (2007), a meta-synthesis is more than a summary. By understanding the connection between the findings, which can be reflected in a concept, a metaphor, or a theory, one can achieve a synthesized understanding of a subject or experience that may inform actual policy makers and clinical practitioners (Finfgeld, 2003).

### Research Design

Following the approach of Sandelowski and Barroso (2007), we performed a metasummery (aggregation of the findings and calculation of their frequency across the studies) and a meta-synthesis (defining categories and arriving at a third order interpretation). We adhered to the five steps suggested by Sandelowski and Barroso: formulating purpose and background; searching for and selecting relevant qualitative studies; critically appraising the detected articles; categorizing the articles’ findings, aggregating and synthesizing the research findings; and arriving at a novel interpretation (third order construct) of participants’ quotes (first order constructs) and the original authors’ interpretations (second order constructs) in the primary studies.

### Search Strategy

Based on a research protocol published in Prospero in 2023 (CRD42023379068), three of the authors (TS, GMH & GK) performed systematic searches in the five databases CINAHL, MEDLINE, EMBASE, Sociological Abstracts and PsycINFO between January and Mars 2023, guided by an experienced university librarian. The searches were updated in November 2024. The search strategy was adapted to the different databases using combinations of thesaurus/subject headings/MESH terms and free text searches. We used various combinations of search terms (Table 1). Additionally, TS, GMH & GK performed ancestry searches in the reference lists of included studies and forward tracking using the “cited by” function in Google Scholar. TS & GK also checked the reference lists of recently published literature reviews, and we performed searches in some journals: *Journal of Men’s studies, Men and Masculinities, PFLEGE and Qualitative Health Research.*

**Table 1.** Search Terms.

(human male* OR man OR men OR husband* OR spouse* OR partner* OR caregiver* OR carer* OR family OR families OR couple*)	**AND**	(dementia OR alzheimer’s disease OR alzheimer OR cognitive dysfunction)	**AND**	(at home OR home OR homecare OR home environment)	**AND**	(experience* OR attitude* OR duty OR duties OR responsibilit* OR caring behavior* OR caring behaviour*OR role*OR perspective* OR caregiver strain OR caregiver burden OR caregiver attitude OR interview* OR qualitative OR narrative)

### Inclusion Criteria

We included peer-reviewed qualitative studies that explore the experiences of male caregivers providing care to a female spouse or partner living with dementia at home. To ensure accurate interpretation and analysis, only articles published in English, Norwegian, Swedish, Danish, or German—languages in which the authors have strong proficiency—were considered. Studies published between 2000 and 2024 were included, a time frame chosen to limit the number of articles and allow for a more in-depth analysis (Noblit & Hare, 1988). Additionally, we mapped studies published prior to 2000 to ensure that key aspects of the phenomenon under investigation were captured, as recommended by Booth et al. (2022).

### Exclusion Criteria

Scientific articles published before the year 2000, articles written in languages other than those specified above and articles that exclusively focus on men’s experiences of performing household chores, and articles in which most of participants were caregivers other than spouses or partners.

### Study Selection

**Figure 1.**
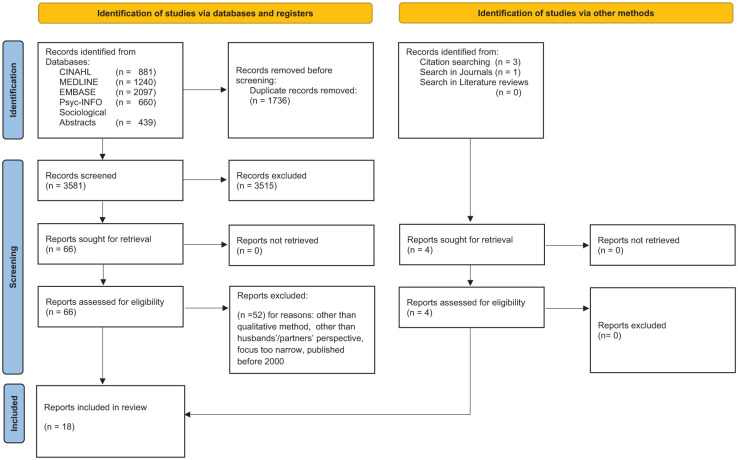
Prisma 2020 flow diagram. *Source*. Page et al. (2021).

Three of the authors collaborated (TS, GMH, GK) to screen the titles and abstracts of the identified studies against the inclusion criteria (Figure 1). All authors participated in the independent screening of the full texts, working in pairs and discrepancies were discussed within the research team. Eighteen articles were included.

### Critical Appraisal

The eighteen studies were assessed independently by three authors (TS, GMH, GK) using the Critical Appraisal Skills Programme (CASP) checklist for qualitative studies (CASP, 2023). This tool is one of the most used appraisal tools in meta-synthesis research (Majid & Vanstone, 2018). The research team concluded that the overall quality of all 18 studies was satisfying, providing valuable data for our meta-synthesis (Table 2).

**Table 2.** Critical Appraisal of Studies (CASP).

First author (year)	Was there a clear statement of the aims for the research?	Is a qualitative methodology appropriate?	Was the research design appropriate to address the aims of the research?	Was the recruitment strategy appropriate to the aims of the study	Was the data collected in a way that address the research issue?	Has the relationship between researcher and participants been adequately considered?	Was the data analysis sufficiently rigorous?	Have ethical consideration been taken?	Is there a clear statement of findings?	How valuable is the research?	Score
Bauernschmidt and Dorschner (2014)	Y	Y	Y	Y	Y	CT	Y	Y	Y	Y	9/10
Black et al. (2009)	Y	Y	Y	Y	Y	CT	Y	Y	Y	Y	9/10
Calasanti and King (2007)	Y	Y	Y	Y	Y	CT	Y	CT	Y	Y	8/10
Hellström et al. (2017)	Y	Y	Y	Y	Y	CT	Y	Y	Y	Y	9/10
Kirsi et al. (2000)	Y	Y	Y	Y	Y	Y	Y	Y	Y	Y	10/10
Kirsi et al. (2004)	Y	Y	Y	Y	Y	Y	Y	Y	Y	Y	10/10
Knutsen and Råholm (2009)	Y	Y	Y	Y	Y	CT	Y	Y	Y	Y	9/10
Pretorius et al. (2009)	Y	Y	Y	Y	Y	CT	Y	Y	Y	Y	9/10
Russell (2001)	Y	Y	Y	Y	Y	CT	CT	Y	Y	Y	8/10
Russell (2007a)	Y	Y	Y	Y	Y	CT	CT	Y	Y	Y	8/10
Russell (2007b)	Y	Y	Y	Y	Y	CT	CT	Y	Y	Y	8/10
Rykkje and Tranvåg (2019)	Y	Y	Y	Y	Y	Y	Y	Y	Y	Y	10/10
Sampson and Clark (2016)	Y	Y	Y	Y	Y	CT	Y	Y	Y	Y	9/10
Sanders and Power (2009)	Y	Y	Y	Y	Y	Y	Y	Y	Y	Y	10/10
Simpson et al. (2018)	Y	Y	Y	Y	Y	Y	Y	Y	Y	Y	10/10
Stefánsdóttir et al. (2022)	Y	Y	Y	Y	Y	Y	Y	Y	Y	Y	10/10
Tolhurst and Weicht (2023)	Y	Y	Y	Y	Y	Y	Y	Y	Y	Y	10/10
Zhang (2021)	Y	Y	Y	Y	Y	CT	CT	CT	Y	Y	7/10

*Note*. CT = can’t tell; Y = yes; N = no.

### Ethical Considerations

No ethical approval was needed due to the use of published data.

### Rigor

We applied two mechanisms recommended by Sandelowski and Barroso (2007) to establish rigor: a detailed audit trail documenting all research procedures and negotiated consensual validity, meaning decisions were based on thorough discussions and consensus within the research team.

### Metasummery

We performed a metasummary in which findings were abstracted, grouped, and effect sizes were calculated to verify how categories are represented and weighted (Sandelowski & Barroso, 2007) (Table 3).

**Table 3.** Effect Sizes—Metasummary of Intrastudy Effect Sizes and Calculated Interstudy Frequency Sizes of Category.

Categories						
Author (year)	Getting used to a new normal—the transition from husband to caregiver	A commitment and moral duty to care	Trying to deal with spouses’ cognitive decline and deviant behavior—an emotional turmoil	Grieving their losses	Embracing a caregiver identity	Intrastudy intensity effect size (%)
Bauernschmidt and Dorschner (2014)	X	X	X	X	X	100
Black et al. (2009)	0	X	X	X	X	80
Calasanti and King (2007)	X	X	X	X	X	100
Hellström et al. (2017)	X	X	X	X	X	100
Kirsi et al. (2000)	X	X	X	X	X	100
Kirsi et al. (2004)	X	X	X	X	X	100
Knutsen and Råholm (2009)	X	X	X	X	X	100
Pretorius et al. (2009)	X	X	X	X	X	100
Russell (2001)	X	X	X	X	X	100
Russell (2007a)	X	X	X	X	X	100
Russell (2007b)	X	X	X	X	X	100
Rykkje and Tranvåg (2019)	X	X	X	X	X	100
Sampson and Clark (2016)	X	X	X	X	X	100
Sanders and Power (2009)	X	X	X	X	X	100
Simpson et al. (2018)	X	X	X	X	X	100
Stefánsdóttir et al. (2022)	X	X	X	X	X	100
Tolhurst and Weicht (2023)	X	0	X	X	X	80
Zhang (2021)	X	X	X	X	X	100
Interstudy frequency effect sizes (%)	94.4	94.4	100	100	100	

### Meta-Synthesis

To analyze the datasets in the primary studies we created a data extraction sheet containing columns for the participating men’s quotes (first order constructs), the primary researchers’ interpretations (second order constructs), and finally a column for the location of constructs in the studies included. Each of the 18 studies’ first and second order constructs were transferred to a separate sheet. According to Sandelowski and Barroso (2007), it is crucial to localize “in vivo” concepts and metaphors that illuminate the essential meaning of the studies’ findings. All authors participated in the extraction of data and identified concepts, metaphors and symbols conveying the meaning of being a male caregiver. The core findings were subsequently collected and systematically compared with careful attention to the underlying meaning. We used reciprocal translation analysis (Noblit & Hare, 1988), one of the analysis methods recommended for the use in meta-synthesis (Sandelowski & Barroso, 2007). Reciprocal translation was possible as the main concepts of the studies were similar and could be translated into each other (Noblit & Hare, 1988). Subsequently, the core findings were abstracted into three main categories and four subcategories. Finally, we developed an overarching metaphor derived from the synthesis process to foster a deeper understanding of the phenomenon under investigation.

## Results

The findings are reported as a metasummary and a meta-synthesis.

### Metasummary

Eighteen studies from nine different countries published between 2000 and 2023 were included. The total sample size comprised 226 male caregivers (interviews: *n* = 200, written narratives: *n* = 26). In most of the included studies, participants were aged 65 and older, with the majority falling within the 70–85-year age range (Table 4).

**Table 4.** Characteristics of Included Studies.

	**First author (year), country**	**Aim**	**Research design**	**Data collection method**	**Sample**	**Data analyses**	**Main results**
**1**	Bauernschmidt and Dorschner (2014), Germany	To gain insight how husbands experience taking care of their wives with dementia at home	Interpretative phenomeno-logical approach	Narrative interviews	10 German husbands, Age 67–95 years (*M* = 75) Length of marriage: 22–67 years (*M* = 49) Time range of caregiving: 1, 5–30 years (*M* = 8.5)	Modified phenomeno-logical interpretative approach according to Diekelmann (1992)	Seven central issues evolved: The disease as a lingering crisis; relationship, commitment; conflict; loss; increasing care burden; limitations in daily life; support
**2**	Black et al. (2009), USA	To explore experiences of suffering in late life of oldest-old male caregivers when caring for their wives with dementia	Ethnographic approach—constructivist framework	Formal ethnographic interviews and informal conversation	4 American husbands, Age 80 and above Further details on the sample are missing	Qualitative data analysis according to Mischler (1986) and Silverman (2001)	Three thematic “tools” of control emerge as strategies the men used to mediate their suffering: (1) The power of the little. (2) Preserving Self-Identity and Marriage-Identity. (3) Finding purpose in the role of caring.
**3**	Calasanti and King (2007), USA	To explore husbands’ experiences of caring for wives with Alzheimer disease	Constructivist, feminist structural approach	In-depth interviews and observations in support groups.	9 American husbands 65–83 years (*M* = 72) Length of marriage: 28–58 years	Iterative process, data were coded by the research team using QSR NUD*IST	Identified six strategies husband used to deal with caregiving problems: – exerting force, – focus on tasks, – blocking emotions, – minimizing disruption – distracting attention, – self medicating
**4**	Hellström et al. (2017), Sweden	To describe how older Swedish men approach the caregiver role of a wife with dementia, over time	Qualitative approach Secondary analysis	Semi-structured interviews	7 Swedish husbands interviewed up to five occasions Age 71–85 years Length of marriage: 8–60 years Memory problems up to approximately 3 years	Thematic analysis	Three themes were revealed: – Me and it—*a distance between the self- and the care practices.* – Me despite it—*caregiving moves closer.* – It is me—*being a caregiver man.*
**5**	Kirsi et al. (2000), Finland	To interpret the husbands’ stories as caregivers into texts in their one right; how they assign meanings and make sense of their lives as caregivers	Qualitative approach	15 narratives written by Finnish men	15 narratives written by Finnish husbands Further details on the sample are missing	Discourse analysis	There is a tension between being a caregiver and being a man. The main finding was the diversity of husbands’ experiences of caregiving and the contextuality of the ways in which these experiences were talked about.
**6.**	Kirsi et al. (2004), Finland	To focus on husbands’ descriptions of their everyday experiences of caregiving	Qualitative approach	Written texts and interviews	11 free form written texts by Finnish husbands, and 13 focus interviews with Finnish husbands Further details on the sample are missing	Constructive interpretation of the husband’s text and talk.	The result of the study challenge interpretation of men as eighter ineffective or capable caregivers and highlight, instead, the contextual nature of the way that men construct their agencies, depending upon the purposes and audiences of their narration.
**7**	Knutsen and Råholm (2009), Norway	To understand the experiences of male caregivers taking care of their wives suffering from dementia	Phenomeno-logical study	Individual interview	9 ethnic Norwegians husbands Age 65–87 years (*M* = 79.5) Time range of caregiving: 3–7 years	The analysis followed the four steps derived from Giorgi	The essence of the men’s experiences is a daily struggle with grief, loneliness while experiencing reconciliation through love and communion.
**8**	Pretorius et al. (2009), South Africa	To obtain a picture of the psychological experiences of men caring for a spouse diagnosed with dementia within the South African context	Qualitative part of mixed methods approach	Semi-structured interviews	10 husbands Age: 61–68 years (*M* = 77) Time range of caregiving: 1–23 years one English speaking individual 9 Afrikaans-speaking individuals	An orientational analysis using Antonovsky`s sense of coherence as a theoretical basis,	Caregivers were exposed to numbers of stressors, most often reported: cognitive impairment, behavior problems, lack of free-time, erosion of the relationship, family conflict and financial concern.
**9**	Russell (2001), USA	To explore the richness and diversity of experiences encountered by elderly men caregivers and the variety of meanings to their caregiving experiences	Qualitative approach	Open-ended Interviews	14 husbands Age 68–90 years One African, two recent Eastern European immigrants Further details on the sample are missing	Inductive analysis	Three major themes: Feelings of isolation and invisibility of their care work A style of caregiving that jointly utilizes management and nurturing Feelings of commitment, responsibility and devotion
**10**	Russell (2007a), USA, Men Doing “Women’s Work:” Elderly Men Caregivers and the Gendered Construction of Care Work	To investigate two specific areas of care work: meal preparation and personal care, as well as the subjective meanings ascribed to such work	Qualitative approach	Open-ended interviews	30 elderly husbands Details on the sample are missing	The transcripts were coded, and the emerging themes and concepts were evaluated	Many struggled with the demands of care work, especially within entrenched gender norms and masculine scripting. Men are less avoidant of hands-on, personal care that has been reported in the past
**11**	Russell (2007b), USA, The Work of Elderly Men Caregivers, *From Public Careers to an Unseen World*	To explore how elderly men caregivers adapt to the transition from work in the public area to the private, largely invisible world of family care and what resources they call upon to ameliorate the impact of those changes and what can be learned from their experiences	Qualitative approach	Open-ended interviews	30 elderly husbands Age 68–90 years one African, two Eastern European immigrants Further details on the sample are missing	The transcripts were coded, and the emerging themes and concepts were evaluated	Two major themes: The work nobody sees (struggling to cope with the isolation of working in the home environment and the invisibility of care work Joint management/Nurturing style of caregiving (successfully making the transition through a caregiving style combining management and nurturing skills)
**12**	Rykkje and Tranvåg (2019), Norway	To explore the experiences of husbands living at home with their spouse with dementia	Qualitative exploratory, hermeneutic approach	Interviews	5 ethnic Norwegian husbands Age 72–82 years Length of caregiving: max. 3 years	Hermeneutical analysis according to Fleming et al. (2003)	Four subthemes were identified as aspect of managing everyday life: Adapting through openness Living with change, loss, and bereavement Redefining personal freedom Expanding areas of responsibility
**13**	Sampson and Clark (2016), England	To uncover the nuanced details of decision making in practise of men providing care who for females with dementia	Qualitative approach	Semi-structured interviews	7 White British husbands 1 son 1 nephew Age (husbands) 60–90 years Further details are missing (Only the accounts of husbands are included in our meta-synthesis)	Thematic analysis	Two interlinked themes: Caring day to day Reflecting and evaluating decision making (day-to-day decisions, medium-term decision-making, longer-term decision making) The carers adopt a form of practised autonomy to negotiate the complexity of everyday decision making while managing longer term uncertainty and anxiety
**14**	Sanders and Power (2009), USA	To examine the changes that occurred in the roles, responsibilities, and relationships of husbands providing care for their wives	Qualitative approach, phenomeno-logy	Semi-structured interviews	17 American husbands (all Whites) were interviewed twice with approximately 4 months apart Age 66–85 years (*M* = 77) Length of marriage: 30–62 (*M* = 47 years)	Data analysis phenomeno-logical perspective	Two main themes: Adaption of old roles to new roles due to increased responsibility (including 4 categories) Developing new relationships with their wives (including 4 categories)
**15**	Simpson et al. (2018), Alabama, USA	To explore male caregivers’ perceptions of their caregiving roles and their perceptions of a support group-specifically	Qualitative descriptive approach	Semi-structured Interviews	6 Caucasian husbands Age 74–85 years Length of caregiving: 1–5 years	Thematic analysis	Two main themes with 5 subthemes Caregivers’ perceptions of experience: Loss of golden years (loss of wife, family and friends, loss of self) Benefits and improvements of support groups: Creating our own space (Releasing our frustrations; Gendered experience of caregiving)
**16**	Stefánsdóttir et al. (2022), Iceland and Norway	To shed light on couple hood changes as experienced by men caring for wives with dementia	Constructivist grounded qualitative approach	In-depth Interviews	8 husbands from Norway and Iceland Age 67–92 years (*M* = 84) Length of caregiving: 4–10 years	Constant comparative method	Four separate phases/themes in the caring process were identified: (1) the denial phase, (2)the battle phase, (3) the new reality phase, (4) the redefinition phase
**17**	Tolhurst and Weicht (2023), England	To explore how couples negotiate relationships and care following a diagnosis of dementia	Qualitative gender approach	Semi-structed couple interviews	10 couples (all British Whites) were interviewed twice with a 6-month interval Age 62–86 years Length of marriage: 5–58 years Time since diagnosis: 3 months to 9 years	Thematic analysis	Three principal themes were identified: Making sense of the condition Treating dementia as a problem to be solved. Engaging with professionals and support
**18**	Zhang (2021), China	To examine motivation, practices, struggles and strategies of male caregivers	Ethnographic approach	In-depth interviews	60 male Chinese caregivers (40 husbands and 20 sons) Age of husbands 51–90 (*M* = 76) Further details are missing (Only the accounts of husbands were included in our meta-synthesis)		Husbands who engage in caregiving are motivated by love, affection, moral obligation, reciprocity based on past assistance. The expanding home roles of male caregivers call attention to the transformation of gendered care practice in China and beyond

### Meta-Synthesis

The synthesis revealed three main categories (From Partner to Caregiver: Adjusting to a New Normal; From Connection to Solitude: Navigating the Loss of Companionship and Social Participation; Becoming a Caregiver: The Journey Toward Embracing a Caregiver Identity), and four subcategories (Caregiving as a Full-Time Commitment; A Commitment and Moral Duty to Care; Dealing with Spouses’ Cognitive Decline and Deviant Behavior—An Emotional Turmoil; Grieving their Losses) and an overarching metaphor: “Seeking a bridge over troubled waters.” Overall, the findings highlighted the older men’s deep concern about the profound changes in their daily lives resulting from their partners’ cognitive decline. Three major transitions were constructed based on the older men’s descriptions of caring for a partner living with dementia—marking a significant shift in their roles, responsibilities, and sense of self. The sequence of categories was guided by the progression of cognitive symptoms in the ill partner, which led to increasing responsibilities and emotional strain for the caregivers. The emergence of a caregiver identity was viewed as a gradual, long-term process and is therefore presented as the final category.

### From Partner to Caregiver: Adjusting to a New Normal

Across all studies, it was consistently found that when the ill partner could no longer manage activities of daily life, caregiving became a more prevalent aspect of men’s lives. The transition from being caring husbands to becoming spousal caregivers brought about radical changes and complex emotions related to unfamiliar caregiving tasks. The following text presents two subcategories that illustrate how caregivers experienced the transition from partner to caregiver, and their motivations for taking on caregiving responsibilities.

#### Caregiving as a Full-Time Commitment

Assisting with dressing and intimate care, particularly managing incontinence, was challenging for many (Bauernschmidt & Dorschner, 2014; Calasanti & King, 2007; Kirsi et al., 2000, 2004; Pretorius et al., 2009; Russell, 2007a; Sanders & Power, 2009; Stefánsdóttir et al., 2022). One man described the emotional toll bluntly: “. . . for a man it is very difficult . . . I have overcome my disgust, but it is terrible . . .” (Bauernschmidt & Dorschner, 2014, p. 301).^1^ Others perceived assisting with personal care as a natural part of the caregiving role and were surprised by how quickly they adapted (Calasanti & King, 2007; Kirsi et al., 2004; Pretorius et al., 2009; Sanders & Power, 2009).

When the ill partner could no longer manage household chores, the men struggled with these tasks (Bauernschmidt & Dorschner, 2014; Kirsi et al., 2004; Russell, 2001, 2007a; Sampson & Clark, 2016; Sanders & Power, 2009; Simpson et al., 2018; Stefánsdóttir et al., 2022). One participant expressed growing frustration over the imbalance in household responsibilities, stating: “I’m doing everything now. . . she doesn’t do anything [. . .]^2^ I cook breakfast, do the housecleaning and wash everything. I’m pretty frustrated” (Simpson et al., 2018, p. 314). Caring for their wives 24/7 was exhausting and perceived as a full-time job (Russell, 2007a; Simpson et al., 2018; Zhang, 2021).

Some men hired help or applied for home care services (Hellström et al., 2017; Knutsen & Råholm, 2009; Pretorius et al., 2009; Russell, 2001). However, seeking professional assistance was not an option for all caregivers (Sanders & Power, 2009; Simpson et al., 2018). Reasons included pride (Kirsi et al., 2004; Knutsen & Råholm, 2009; Rykkje & Tranvåg, 2019), fear for the wife’s well-being (Bauernschmidt & Dorschner, 2014; Black et al., 2009; Sampson & Clark, 2016; Tolhurst & Weicht, 2023) or the wife’s non acceptance of strangers (Zhang, 2021). Many men longed for more support (Bauernschmidt & Dorschner, 2014; Kirsi et al., 2004; Russell, 2001, 2007a, 2007b; Sanders & Power, 2009; Simpson et al., 2018; Stefánsdóttir et al., 2022; Tolhurst & Weicht, 2023; Zhang, 2021).

During their working lives, men’s productivity and competence had been recognized (Russell, 2007b). Now their work as primary caregivers went unnoticed and remained hidden within the home (Russell, 2007a, 2007b). One man described a growing sense of invisibility: “I never knew how hard this was, because I never saw what she did, and . . . Nobody sees what we’re doing” (Russell, 2007b, p. 303).

Positive experiences with health care workers were rarely mentioned (Knutsen & Råholm, 2009; Pretorius et al., 2009; Russell, 2001). Negative experiences led to mistrust of health services (Knutsen & Råholm, 2009; Russell, 2001; Tolhurst & Weicht, 2023). One man described feeling alienated and misunderstood during interactions with care professionals: “[. . .] It’s not easy to talk about everything with these lasses who have rings in their ears and swoosh around when they come to see her [. . .]. And they talk as if I’m hard of hearing. Sometimes they think I’m stupid too” (Knutsen & Råholm, 2009, p. 52).

#### A Commitment and Moral Duty to Care

The men viewed caregiving as a responsibility towards their wives (Russell, 2007a; Sampson & Clark, 2016; Stefánsdóttir et al., 2022). They felt a profound obligation to care rooted in their marriage vows: *“*until death do us part” (Simpson et al., 2018, p. 313). One participant emphasized a strong sense of moral responsibility in their caregiving role, stating: “I think it is my duty to care because if it had been vice versa, I think she would have looked after me . . .” (Sampson & Clark, 2016, p. 1609).

Men’s motivation to care stemmed from their commitment and loyalty to their ill partner (Black et al., 2009; Kirsi et al., 2000; Russell, 2001, 2007a; Rykkje & Tranvåg, 2019; Sampson & Clark, 2016; Simpson et al., 2018; Stefánsdóttir et al., 2022). They tried to repay their partners for their contributions throughout the marriage (Knutsen & Råholm, 2009; Pretorius et al., 2009; Russell, 2001). Reflecting on his partner’s past contributions to the family, one man expressed a deep sense of gratitude and reciprocity, saying: “She was a good wife. Was there for the children and me. She was a fine mother. . . . It`s good to be able to give something back [. . .]” (Knutsen & Råholm, 2009, p. 53).

Many older men reported a lack of support from their adult children, who were preoccupied with their own lives or lacked a clear understanding of the illness and the challenges associated with caregiving (Bauernschmidt & Dorschner, 2014; Russell, 2001, 2007b; Sanders & Power, 2009; Simpson et al., 2018; Zhang, 2021). One man shared his frustration, stating: “My difficulty is no support! [. . .] I call him [son] and say ‘hey, can you give me a hand with . . .’ They respond, ‘we’ve got plans, we’ve got stuff to do’ and that’s what I hear all the time [. . .]” (Simpson et al., 2018, p. 312). Rejection by their children sometimes led to family conflicts (Russell, 2001, 2007a; Zhang, 2021) exacerbating the emotional turmoil experienced by caregivers who felt alone with responsibility.

Positive responses from the ill partner such as gratitude and caring attitudes strengthened the relationship (Hellström et al., 2017; Knutsen & Råholm, 2009; Zhang, 2021). Some men tried to preserve the marital relationship as long as possible (Black et al., 2009; Simpson et al., 2018). Others felt their relationship had become stronger (Rykkje & Tranvåg, 2019; Tolhurst & Weicht, 2023). A new type of intimacy and closeness developed in some couples (Russell, 2001; Sanders & Power, 2009; Tolhurst & Weicht, 2023). One participant shared how caregiving fostered moments of joy and strengthened emotional bonds: “[. . .] we laugh and joke [. . .] we have a dance in the kitchen [. . .] and it’ s brought us so much closer together you know” (Tolhurst & Weicht, 2023, p. 6).

Despite their spouses’ worsening illness, men hesitated to place them in a nursing home, either due to moral obligation (Stefánsdóttir et al., 2022), fear of loneliness (Stefánsdóttir et al., 2022; Zhang, 2021), concerns about the economic burden on society (Rykkje & Tranvåg, 2019), economic considerations (Russell, 2007a) or uncertainty about care quality (Russell, 2001; Sanders & Power, 2009). However, as the illness progressed, some men had to apply for placement in a nursing home (Bauernschmidt & Dorschner, 2014; Russell, 2007b; Stefánsdóttir et al., 2022).

### From Connection to Solitude: Navigating the Loss of Companionship and Social Participation

The older men reflected on the shift in marital quality before and after the onset of dementia. The loss of companionship marked a painful transition, evoking a range of emotions, as described in the first subcategory. The second subcategory highlights how being homebound and constantly monitoring the ill partner restricted social engagement, leaving the older men feeling lonely and isolated.

#### Dealing with Spouses’ Cognitive Decline and Deviant Behavior—An Emotional Turmoil

In all studies, the cognitive decline of the spouse, coupled with communication problems and challenging behavior, increased the demand placed on male caregivers’ lives. Some battled guilt and the perceived moral obligation to provide support:” [. . .] I struggle with my bad conscience because I got irritated and angry with her. I’m so tired . . . these experiences are very hard for me to admit” (Knutsen & Råholm, 2009, p. 53). Aggressive and unpredictable behavior caused anger, resentment and guilt, making caregivers feel that they were married to a stranger (Bauernschmidt & Dorschner, 2014; Calasanti & King, 2007; Kirsi et al., 2000; Rykkje & Tranvåg, 2019; Sampson & Clark, 2016; Sanders & Power, 2009; Stefánsdóttir et al., 2022). One husband described how a dramatic incident impacted his role as husband, neighbor and caregiver: “[. . .] One day she went over to the next-door neighbor’s and wanted to whip and kill us both. I had to walk out of there and lock myself in the car. She picked up a big rock and threw it on the windshield. The police had to come. [. . .]” (Sanders & Power, 2009, p. 48).

Interfering with their partner’s autonomy, such as locking doors, was difficult (Calasanti & King, 2007; Kirsi et al., 2004; Pretorius et al., 2009). Some men acknowledged having used force or spoken harshly to their partners during moments of feeling overwhelmed and helpless: “I remember bruising her once on her arm and ever since then, boy, that is embarrassing to leave a bruise” (Calasanti & King, 2007, p. 523). Others remained silent to avoid conflicts, relying on their partner’s short memory. The ill partner’s lack of response or repetitive behavior was frustrating and caused shame and guilt when caregivers raised their voices (Kirsi et al., 2004; Pretorius et al., 2009; Rykkje & Tranvåg, 2019).

As their partners’ cognitive decline progressed, many older men expressed growing concern for their partners’ safety (Sampson & Clark, 2016) and their own ability to cope as the illness progressed (Bauernschmidt & Dorschner, 2014; Black et al., 2009; Hellström et al., 2017; Zhang, 2021). Thinking about how their ill partners might cope alone filled the men with fear for the future: “I worry about that sometimes, how the hell will she manage because she is incredibly dependent on me, so if I should disappear, then . . .” [crying] (Hellström et al., 2017, p. 961).

Feelings of inadequacy in fulfilling caregiving obligations often resulted in guilt (Kirsi et al., 2004; Knutsen & Råholm, 2009; Russell, 2007b; Tolhurst & Weicht, 2023). The men felt exhausted and deprived of sleep (Knutsen & Råholm, 2009), felt ill (Kirsi et al., 2004; Zhang, 2021), or sought solace in alcohol to escape reality (Bauernschmidt & Dorschner, 2014; Calasanti & King, 2007).

#### Grieving Their Losses

Living with grief, loss and unpredictability due to their partners’ cognitive decline was hard for the older men (Black et al., 2009; Hellström et al., 2017; Kirsi et al., 2000; Rykkje & Tranvåg, 2019). Accepting the harsh reality that there was no hope for a cure proved deeply difficult for the men: “[. . .] but I have to resign myself that she will never be back with me” (Sampson & Clark, 2016, p. 1612). Losing valued activities and meaningful conversations with their wives was tough (Knutsen & Råholm, 2009; Sanders & Power, 2009).

Loss of intimacy and sexual activity was also challenging to reconcile and cope with (Kirsi et al., 2004; Russell, 2007a; Sanders & Power, 2009; Stefánsdóttir et al., 2022). One man reflected on the emotional impact of losing physical and sexual intimacy in his relationship, describing feelings of rejection, longing, and grief as his partners became less responsive to affection: “There wasn’t the love times when we would caress and stuff. I tried to and I could see she just lost interest. I miss the sex . . . sexual intimacy [. . .]” (Sanders & Power, 2009, p. 47).

Their wives had always been their most important source of support, as the illness progressed the older men struggled to find support elsewhere (Knutsen & Råholm, 2009; Russell, 2001; Simpson et al., 2018; Stefánsdóttir et al., 2022; Zhang, 2021). They no longer recognized the person their spouse had become (Bauernschmidt & Dorschner, 2014; Knutsen & Råholm, 2009; Russell, 2001; Rykkje & Tranvåg, 2019; Sanders & Power, 2009; Stefánsdóttir et al., 2022). A husband described the emotional weight of change—not just in their partner’s condition, but in the relationship itself: “[. . .] like losing one’s spouse . . . that is how I feel. You have a completely different person in your hands that you do not know. You treat her entirely differently and interact with her differently. There is obvious affection and all that. It is just a completely different landscape” (Stefánsdóttir et al., 2022, pp 748).

Caregiving 24/7 took a toll on the men. Finding no escape and feeling tied to their home brought forth strong feelings of isolation and loneliness (Bauernschmidt & Dorschner, 2014; Black et al., 2009; Calasanti & King, 2007; Hellström et al., 2017; Kirsi et al., 2000, 2004; Knutsen & Råholm, 2009; Russell, 2001; Rykkje & Tranvåg, 2019; Sanders & Power, 2009; Simpson et al., 2018; Stefánsdóttir et al., 2022; Tolhurst & Weicht, 2023). One man captured the emotional toll of caregiving succinctly: “Being a carer. . . it’s very lonely existence . . .” (Tolhurst & Weicht, 2023, p. 9). The sense of loneliness deepened for some men if their wives no longer recognized them (Kirsi et al., 2000; Knutsen & Råholm, 2009; Sanders & Power, 2009), as one man powerfully expressed: “[. . .] I wasn’t her husband anymore. I was someone else [. . .]” (Knutsen & Råholm, 2009, p. 53).

The men mourned the loss of their wives as they once knew them (Simpson et al., 2018). Yet, mourning felt unfair as their partner was still alive (Knutsen & Råholm, 2009; Stefánsdóttir et al., 2022), as reflected in this man’s word: “It’s a rare kind of grief because of the circumstances. She’s there. But at the same time, she’s not there. And that’s what the others are unable to see [. . .]” (Knutsen & Råholm, 2009, p. 52). Some felt that a final goodbye would have been easier—and a relief for both—compared to the gradual loss of their partner over time (Kirsi et al., 2004). Whereas others expressed gratitude that their wives were still with them and had not been lost to sudden death (Knutsen & Råholm, 2009).

Their wives’ grief about their condition amplified the men’s grief: “This is suffering because it doesn’t get better. It gets deeper and deeper. And you know it, and not only do you know it, but she knows it” (Black et al., 2009, p. 189). Venting such feelings was hard (Calasanti & King, 2007; Sanders & Power, 2009; Simpson et al., 2018; Stefánsdóttir et al., 2022). Allowing themselves to grieve was seen as weakness: “I know I am losing her, and there’s once in a while where you feel sorry for yourself and allow yourself to grieve. Brief moments of weakness” (Sanders & Power, 2009, p. 49). Some men avoided thinking about their challenging situation: “I guess I have built kind of a wall between the sorrow and what has to be done in trying to live with it. I mean I can’t go around feeling horrible all the time [. . .]” (Calasanti & King, 2007, p. 523).

The older men mentioned other losses due to their burden of caregiving, such as the loss of their golden years as pensioners (Simpson et al., 2018), loss of predictability (Simpson et al., 2018) and autonomy (Rykkje & Tranvåg, 2019), loss of future possibilities, and loss of network and appreciated activities (Simpson et al., 2018; Stefánsdóttir et al., 2022).

Fearing the future loss of their wives’ companionship, some male caregivers hesitated to pursue nursing home placement (Bauernschmidt & Dorschner, 2014; Russell, 2001, 2007b; Stefánsdóttir et al., 2022; Zhang, 2021). Imagining the loss of one’s home as it had been before was hard to deal with: “. . . Our home will no longer be a home if she is absent . . . I need her more than she needs me” (Zhang, 2021, p. 10).

### The Journey Toward Embracing a Caregiver Identity

Gradually, the caregivers shifted from viewing caregiving as an unfamiliar task to embracing it as an integral part of their identity, adapting to and accepting the role of caregiver over that of husband.

The older men used problem focused and task-oriented strategies to cope with their roles (Calasanti & King, 2007; Hellström et al., 2017; Pretorius et al., 2009; Stefánsdóttir et al., 2022; Tolhurst & Weicht, 2023). They concentrated on the present, taking one challenge at a time (Black et al., 2009; Russell, 2001; Sampson & Clark, 2016; Stefánsdóttir et al., 2022; Tolhurst & Weicht, 2023). One husband described how focusing on the present helped him cope with daily life: “One day at a time approach, you just get up in the morning, pray for that day. Yesterday is gone and you cannot worry about tomorrow, because it is not here yet’’ (Russell, 2001, p. 363). Some viewed caregiving as a job (Hellström et al., 2017; Rykkje & Tranvåg, 2019; Sampson & Clark, 2016; Simpson et al., 2018). In expressing his sense of duty, a husband shared that resigning was never an option, no matter how demanding caregiving became: “A man takes his caregiving role as a job and you don’t quit a job [. . .]” (Simpson et al., 2018, p. 315). Organizing their day to maintain control and ensure predictability was a priority (Calasanti & King, 2007; Hellström et al., 2017; Kirsi et al., 2004; Knutsen & Råholm, 2009; Pretorius et al., 2009; Sampson & Clark, 2016). Despite challenges, they did what was necessary without much reflection (Calasanti & King, 2007; Sampson & Clark, 2016).

Some men continued former activities providing them with a source of energy and stability (Calasanti & King, 2007; Kirsi et al., 2000; Pretorius et al., 2009; Russell, 2001; Sanders & Power, 2009). Others used humor as a coping strategy (Pretorius et al., 2009). For pensioners, caregiving replaced the breadwinner role (Russell, 2007a; Sampson & Clark, 2016; Zhang, 2021). Managerial skills from work were useful (Russell, 2007a, 2007b), and those still working valued the variety (Pretorius et al., 2009; Russell, 2001). Staying connected with the world (Black et al., 2009; Pretorius et al., 2009), finding comfort in religion (Black et al., 2009; Kirsi et al., 2000; Pretorius et al., 2009), engaging in voluntary work (Kirsi et al., 2000; Simpson et al., 2018), or participating in peer groups (Simpson et al., 2018; Tolhurst & Weicht, 2023) was important. Respite care was appreciated by some (Calasanti & King, 2007; Kirsi et al., 2000; Russell, 2001, 2007b; Sampson & Clark, 2016; Stefánsdóttir et al., 2022).

Keeping spirits up was crucial for dealing with challenges (Bauernschmidt & Dorschner, 2014; Black et al., 2009; Stefánsdóttir et al., 2022). One participant expressed his acceptance of the limitation caregiving placed on his lifeworld: “The world is shrinking . . . and in this little world you have to try. . . finding it beautiful, to make something out of it: thinking: it is good as it is” (Bauernschmidt & Dorschner, 2014, p. 301). Understanding and accepting the illness trajectory helped manage spouses’ cognitive decline and deviant behavior (Rykkje & Tranvåg, 2019; Sampson & Clark, 2016; Simpson et al., 2018; Tolhurst & Weicht, 2023). One husband reflected on the emotional adjustment required in caregiving, expressing his willingness to adapt: “I realize that life has changed. . . and I must accept that . . . I do not regret our situation. I have just “put life on hold” in fact [. . .] I must try to adjust . . . that is how I feel” (Rykkje & Tranvåg, 2019, p. 5).

Over time, the older men adapted to new routines, expressing pride, satisfaction, and confidence when managing tasks and developing new skills (Bauernschmidt & Dorschner, 2014; Calasanti & King, 2007; Hellström et al., 2017; Kirsi et al., 2004; Knutsen & Råholm, 2009; Pretorius et al., 2009; Russell, 2001, 2007a; Rykkje & Tranvåg, 2019; Sampson & Clark, 2016; Sanders & Power, 2009; Tolhurst & Weicht, 2023). The following quote shows the participant’s growing sense of fulfillment in his caregiving role: “[. . .] I like the satisfaction of caring for her [. . .] building up the things I was doing – things I never thought I would be able to do [. . .]” (Sampson & Clark, 2016, p. 1610). Caregiving became part of the men’s identity (Calasanti & King, 2007; Hellström et al., 2017; Russell, 2001, 2007a; Sanders & Power, 2009; Zhang, 2021) as expressed by this man: “. . . But it’s [caregiving] changed . . . it’s something that’s kind of become part of me” (Russell, 2007a, p. 12).

Some men felt they contributed to society (Rykkje & Tranvåg, 2019) and presented themselves as competent carers (Kirsi et al., 2004; Knutsen & Råholm, 2009). Though not in line with the traditional male role, over time, caregiving was perceived as meaningful (Bauernschmidt & Dorschner, 2014; Black et al., 2009; Kirsi et al., 2004; Knutsen & Råholm, 2009; Pretorius et al., 2009; Russell, 2001, 2007a; Zhang, 2021). Some men expressed how caregiving was rooted in care and emotional connection rather than obligation: “[. . .] you get used to these things when you know it isn’t only a duty, but you take care [. . .] you wish that she is thriving. I feel all right if she feels good” (Bauernschmidt & Dorschner, 2014, p. 301). For some men, caregiving provided a new purpose in life (Black et al., 2009; Kirsi et al., 2000; Sampson & Clark, 2016). One man voiced his gratitude for the insight he had acquired: “It is only now that I am grateful to fate for this chance to care and to do something that I feel has real value” (Kirsi et al., 2000, p. 160).

### The Overarching Metaphor: “Seeking a Bridge Over Troubled Waters”

We developed this metaphor to convey our overarching interpretation of the findings. The men in our study were navigating the turbulent waters of unfamiliar roles and responsibilities, brought on by their partners’ cognitive decline. The phrase “*troubled waters*” reflects the profound and often painful changes in their lives.

These men found themselves in a maelstrom of emotions—grieving the gradual loss of their wives’ health, grappling with guilt when they felt they had failed in their caregiving roles, and struggling to maintain a sense of control. Despite these challenges, they yearned to preserve the emotional bond with their partners for as long as possible, often hesitating to consider nursing home placement.

Finding a safe pathway through the turbulent waters of caregiving can be envisioned through the metaphor of a bridge—representing men’s transitions from husbands to caregivers, offering a possible secure passage from one shore of life to the other. Finding this bridge was difficult for the men as they lacked support from family members and professionals. Out of commitment to their wives, they tried to stay afloat in these troubled waters on a day-to-day basis of existence, trying not to reflect on their worries. Little by little, many older caregivers adjusted to their new reality, discovering a bridge over the troubled waters of caregiving. By embracing new values and the personal growth that emerged from their experiences, they found strength and meaning in their evolving roles.

## Discussion

The overarching metaphor *“seeking a bridge over troubled water”* captures the emotional and practical struggles of the older men overwhelmed by responsibilities and lacking adequate support. The discussion explores three key shifts they experienced throughout their caregiving journey.

### The Transition from Partner to Caregiver

For the older men in our study who were accustomed to traditional gender roles, taking over household chores and providing intimate care presented considerable challenges. Assuming new and unfamiliar roles meant crossing the gender gap (Calasanti & Bowen, 2006). Some caregivers in our meta-synthesis complained about the invisibility of caregiving compared to their former work life. Others used work life competence to deal with challenging tasks or perceived caregiving as a job to be done. In response, many older men attempted to reaffirm their masculine identity by drawing on skills from their educational and professional backgrounds as they took on tasks traditionally viewed as outside typical gender roles. Findings in other studies reveal that caregiving can challenge older men’s sense of masculinity (Milligan & Morbey, 2016) and that they actively try to reshape masculinity through their caregiving roles (Bueno, 2025; Robinson et al., 2014).

Our findings show that the transition from husband to caregiver was accelerated by their wives’ cognitive decline. No longer being recognized by their wives was devastating to the couple’s relationship. Disconnection from the spouse role has been described as an identity crisis, as caregivers associate their own sense of self with their loved one’s lost identity (Nguyen & Levkoff, 2020). Our findings indicate that challenging and sometimes aggressive behavior contributed to emotional distance in the couple’s relationship. Cognitive and behavioral changes may affect emotional closeness, leading to feelings of being married to a stranger (Kitzmüller & Ervik, 2015).

The transition from husband to caregiver also implied loss of sexual relationships. It is known that husbands’ desire for sexual intimacy can be lost due to the partner’s incontinence and poor bowel function or when lack of communication in advanced illness stages affects consent to sexual activity (Fee et al., 2021). Nevertheless, older male caregivers often grieve the loss of sexual intimacy (Fee et al., 2021; Hayes et al., 2009; Robinson et al., 2014).

Our meta-synthesis indicates that the loss of spousal closeness added to men’s challenges. Changes in the spousal relationship due to dementia are described as “the disappearance of a way of being” (Macdonald et al., 2020) and an inevitable part of caregiving (Cross et al., 2018).

Despite their struggles the men in our meta-synthesis showed a strong moral commitment to caregiving out of gratitude and long-lasting emotional bonds with their partner. Similar motivations are described in other studies on spousal caregiving in old age (Bueno, 2025; Greenwood et al., 2019; Musgrave-Takeda et al., 2022; Nguyen & Levkoff, 2020; Ribeiro & Paúl, 2008). Strong pre-dementia marital relationships are vital in supporting continued close relationships as the illness progresses (Hellström et al., 2007). Caregivers in the study of Ribeiro and Paúl (2008) took on caregiving to affirm marital bonds and express gratitude. Helping the spouse is seen as a natural part of the long-term relationship (Musgrave-Takeda et al., 2022) and a way to repay love and support (Nguyen & Levkoff, 2020).

Love and acceptance are sources of strength despite caregiving challenges in old age (Greenwood et al., 2019) and commitment and family unity imply a duty to care (Bueno, 2025; Macdonald et al., 2020). However, feeling a moral duty to care due to gratitude and commitment can prevent caregivers from seeking support and neglecting their own needs. Feeling this obligation despite exhaustion and emotional distress can lead to health problems (Martínez-Santos et al., 2021) and poorer quality of life (Alltag et al., 2019).

### The Shift from Connection to Solitude

The men in our meta-synthesis faced loneliness and isolation due to lack of family support and loss of social activities. Some feared even greater loneliness if separated from their wives due to impending nursing home placement. Loneliness is a common finding in studies describing male caregivers’ experiences (Fee et al., 2023; Mayo et al., 2020; Nel & Board, 2019; Robinson et al., 2014; Willis et al., 2020). Loneliness among older adults is generally acknowledged to be deeply harmful, both emotionally and physically (Kitzmüller et al., 2018) and living with a partner with dementia may reinforce the experience of emotional loneliness. In addition, the older men in our study had difficulties expressing their emotions. According to Kenny et al. (2020) male caregivers’ expectations of remaining strong and carrying their burden silently contributes to their loneliness.

In our meta-synthesis caregivers perceived themselves as tied to caregiving 24/7. This resonates with the interpretation of spousal caregivers as hostages to dementia (Macdonald et al., 2020). However, caregiving also provides constant companionship, which buffers loneliness (Willis et al., 2020).

The men in our study missed close relationships with their adult children who prioritized their own life or struggled with the ill parent’s personality changes, and sometimes anger and family conflicts arose from lack of support. Other studies on dementia caregiving indicate that evolving family dynamics can result in conflicts (Fee et al., 2023; Hendricks-Lalla & Pretorius, 2020; Nel & Board, 2019; Oh et al., 2020; Smith et al., 2022).

Negative experiences with formal support services influenced future trust in these services leaving the men in our study with few sources of support. Help-seeking patterns can depend on former support experiences (Robinson et al., 2014) and factors like values, family closeness, social network, costs, availability, and knowledge (Brown et al., 2007). Reluctance to seek support may relate to masculine identity (Milligan & Morbey, 2016) or a sense of duty and pride (Greenwood & Smith, 2015).

The older men in our study felt guilty when struggling with caregiving. Guilt is reported in several studies on dementia caregiving (Caputo, 2021; Cross et al., 2018; Greenwood et al., 2019). Guilt also arises from thoughts of harm and homicide and from passive death wishes (O’Dwyer et al., 2016). In our study, losing their temper and insulting their wives triggered shame and remorse. The abuse often occurred when their ill partner showed resistance, and the men felt overwhelmed. Caregivers in the study of Shirai et al. (2021) reported physical and emotional exhaustion due to the partners’ resistance to care. Anger, frustration, and fatigue are known as reasons for abuse in dementia caregiving (O’Dwyer et al., 2016).

### The Journey Toward Embracing a Caregiver Identity

In our study, most older men gradually shifted from seeing caregiving as unfamiliar to making it an integral part of their identity. This involved adaptation and acceptance of role and relationship changes and dealing with emotional and practical demands while maintaining their own health and well-being. The caregivers in our meta-synthesis gradually adapted to their new life by developing new skills and gaining confidence in mastering caregiving tasks. Nguyen and Levkoff (2020) emphasize the importance of self-perception strategies for active acceptance and adjustment in dementia caregiving. Strategies like positivity, finding rewards, future planning, willpower, faith, and hope help caregivers manage negative emotions. Likewise, acceptance involves viewing life as a mix of adversity, joy, hope, and growth, distinguishing what is within and out of control (Nguyen & Levkoff, 2020).

Recognition is an important feature of maintaining dignity and self-worth in old age (Clancy et al., 2021). According to Yu et al. (2018) a sense of personal accomplishment and gratification is one of the key domains in the positive aspects of dementia caregiving along with social affirmation. In our meta-synthesis, the older men emphasized the invisibility of their caregiving efforts. Recognizing their substantial contributions could have offered them a sense of empowerment and helped ease their transition from husband to caregiver, affirming their evolving identity in the face of profound change.

### Understanding and Supporting Older Male Caregivers Through the Lens of Transition Theory

The experiences of older male caregivers revealed significant changes, shifts, and transformations that, according to Kralik et al. (2006), are hallmarks of life transitions. To better understand these experiences and how nurses can support them, we found the Transition Theory developed by Meleis et al. (2000) particularly insightful.

Meleis (2010) defines transitions as processes triggered by critical life events, leading individuals from one phase, condition, or status to another. These transitions involve both the process and the outcome of complex interactions between individuals and their environments. How a person perceives a transition—whether as a threat or an opportunity for growth—depends on the context, the specific situation, and the individual’s personal resources and support systems (Meleis, 2010).

In our meta-synthesis, the older men experienced profound changes initiated by their partners’ illnesses and the resulting situational shifts. A lack of guidance and professional support appeared to negatively impact their ability to navigate these transitions. According to Meleis et al. (2000), successful transitions require adequate family and community support, access to healthcare services, and sufficient knowledge and preparedness. Additionally, individuals benefit from guidance to develop the skills needed to navigate their new life circumstances. Transition Theory highlights the significance of awareness and active engagement in managing life transitions (Meleis et al., 2000). In our study, the men needed assistance in recognizing the implications of their partners’ illnesses and in preparing to take on their new caregiving roles effectively.

Nurses can play a pivotal role in facilitating this engagement by providing education about dementia and offering practical guidance to help men prepare for these role changes. Our study found that older men may experience difficulties in expressing their emotions to others. Therefore, it is crucial for nurses to pay close attention to the emotional well-being of older male caregivers and provide appropriate support.

Many of the men reported that their family relationships deteriorated because of the impact of dementia. Meleis et al. (2000) highlights the importance of maintaining positive relationships to remain connected and grounded. Nurses can help mitigate relational disruptions (Schumacher & Meleis, 1994). Early and sensitive interventions might have helped prevent some of the family conflicts described in our meta-synthesis.

Furthermore, Meleis et al. (2000) emphasize the significance of the caregiver–care receiver relationship during illness transitions. Dementia often impairs communication, making it difficult for caregivers to share and reflect on daily changes with their partners. Many men in our study mourned the loss of companionship with their wives. In such cases, emotional support from nurses can help alleviate the psychological burden and emotional turmoil they experience.

The meanings individuals assign to life changes can either facilitate or hinder successful transitions (Meleis, 2010). A sense of purpose and personal growth are key indicators of well-being and successful transitions (Meleis & Trangenstein, 1994; Schumacher & Meleis, 1994). In our meta-synthesis, many caregivers found meaning in their commitment to care and developed a renewed sense of purpose and meaning in life. According to Yu et al. (2018), a strong motivation to provide care is a key predictor of whether positive aspects of caregiving will emerge. Reconstructing a meaningful and valued self-identity is essential for navigating transitions (Kralik et al., 2006). Despite the challenges and lack of support, some of the older men in our study found a bridge over the troubled waters of caregiving by embracing their identity as caregivers, demonstrating resilience and personal transformation.

### Strengths and Limitations

We performed extensive searches in five databases, guided by an expert librarian. Additionally, comprehensive manual searches were conducted. Articles were selected and reviewed by pairs of authors, who collaborated to analyze results and identify key concepts and themes. Reflective discussions helped achieve reliable descriptions. Performing a metasummary before synthesis strengthened credibility (Sandelowski & Barroso, 2007).

Transferability to other male dementia caregivers may be influenced by the engagement level of participants, potentially excluding more isolated individuals whose voices are not represented. Dementia caregiving is dynamic, varying over time and influenced by personal resources and support systems. Behavioral and psychological symptoms differ by illness stage, affecting caregivers’ experiences and coping abilities. Including studies that do not distinguish illness stages can be a limitation.

Cultural differences in studies from different continents can both strengthen and limit transferability. However, cultural variety can illuminate male spousal caregiving and role complexity in dementia care. We consider it a strength that two authors have extensive experience in meta-synthesis research, and three are experienced in dementia family caregiving. Therefore, reflexive discussions on our preunderstandings were essential.

## Conclusion with Recommendations

This study offers new insights into the transitional experiences of older male caregivers of a female partner living with dementia, highlighting their shift from partner to caregiver, the move from connection to solitude, and the emergence of a new caregiving identity. The metaphor “Seeking a bridge over troubled waters” portrays caregiving as a demanding journey, highlighting older men’s resilience and their pursuit of strategies that, for some, led to growth, insight, and meaning.

Nurses play a vital role in supporting these transitions by identifying barriers and resources, offering practical and emotional support, and helping caregivers develop new skills. Guidance on dementia progression, access to respite services, and advice on balancing health and caregiving responsibilities are essential. Outreach services can strengthen professional relationships and reduce caregiver strain, while healthcare workers can support family communication and continuity of care.

Longitudinal qualitative studies may help explore how older male caregivers adapt to the progression of dementia in their partner, identify stage-specific challenges, and inform tailored support strategies for healthier caregiving transitions.

## Notes

1. The German quotes were translated by one of the authors, who is a native German speaker.

2. We used square brackets to indicate where text of minor relevance has been removed from the quotes.
